# Expert opinions on improving coercion data collection across Europe: a concept mapping study

**DOI:** 10.3389/fpsyt.2024.1403094

**Published:** 2024-05-29

**Authors:** Jakub Lickiewicz, Simone Agnes Efkemann, Tonje Lossius Husum, Tella Lantta, Luca Pingani, Richard Whittington

**Affiliations:** ^1^ Department of Psychology, Faculty of Health Sciences, Jagiellonian University Medical College, Krakow, Poland; ^2^ LWL University Hospital Bochum, Ruhr University Bochum, Bochum, Germany; ^3^ Faculty of Health Sciences, Oslo Metropolitan University, Oslo, Norway; ^4^ Department of Nursing Science, University of Turku, Turku, Finland; ^5^ Centre for Forensic Behavioural Science, Swinburne University of Technology, Melbourne, VIC, Australia; ^6^ Department of Biomedical, Metabolic and Neural Sciences, University of Modena and Reggio Emilia, Reggio Emilia, Italy; ^7^ Dipartimento ad Attività Integrata di Salute Mentale e Dipendenze Patologiche, Azienda USL-IRCCS di Reggio Emilia, Reggio Emilia, Italy; ^8^ Centre for Research and Education in Security, Prison and Forensic Psychiatry, St. Olavs Hospital, Trondheim University Hospital, Trondheim, Norway; ^9^ Institute of Mental Health, Norwegian University of Science and Technology (NTNU), Trondheim, Norway

**Keywords:** coercion, mapping methodology, mental health, coercion reduction, health policies

## Abstract

**Introduction:**

Coercion is frequently used in mental health practice. Since it overrides some patients’ fundamental human rights, adequate use of coercion requires legal and ethical justifications. Having internationally standardised datasets to benchmark and monitor coercion reduction programs is desirable. However, only a few countries have specific, open, publicly accessible registries for this issue.

**Methods:**

This study aims to assemble expert opinions regarding strategies that might be feasible for promoting, developing, and implementing an integrated and differentiated coercion data collection system in Europe at national and international levels. A concept mapping methodology was followed, involving 59 experts from 27 countries in generating, sorting and rating strategies regarding relevance and feasibility. The experts were all researchers and/or practitioner members of an EU-COST-Action focused on coercion reduction Fostering and Strengthening Approaches to Reducing Coercion in European Mental Health Services (FOSTREN).

**Results:**

A hierarchical cluster analysis revealed a conceptual map of 41 strategies organized in seven clusters. These clusters fit into two higher-order domains: “Advancing Global Health Research: Collaboration, Accessibility, and Technological Innovations/Advancing International Research” and “Strategies for Comprehensive Healthcare Data Integration, Standardization, and Collaboration.” Regarding the action with the higher priority, relevance was generally rated higher than feasibility. No differences could be found regarding the two domains regarding the relevance rating or feasibility of the respective strategies in those domains. The following strategies were rated as most relevant: “Collection of reliable data”, “Implementation of nationwide register, including data on coercive measures”, and “Equal understanding of different coercive measures”. In analysing the differences in strategies between countries and their health prosperity, the overall rating did not differ substantially between the groups.

**Conclusion:**

The strategy rated as most relevant was the collection of reliable data in the nationwide health register, ensuring that countries share a standard understanding/definition of different coercive measures. Respondents did not consider the feasibility of establishing a shared European database for coercive measures to be high, nor did they envision the unification of mental health legislation in the future. There is some consensus on the most suitable strategies that can be adopted to enable international benchmarking of coercion in mental health settings.

## 1 Introduction

Mental healthcare frequently involves restrictive practices toward clients. These practices are usually justified on the basis that coercion in mental healthcare is intended for the client’s good or to protect them (1). Coercion is defined as the act or practice of using force or threat to persuade a patient to do something. It refers to a range of interventions, from mild acts, such as persuasion, to the most oppressive acts of compulsion, such as restrictive devices (2). Coercion encompasses a broad range of practices in the context of mental health care, characterized by the use of force and threats (3). However, the use of coercion entails challenging ethical situations (4, 5), might cause adverse physical and psychological effects and is an emotional stressor for both individuals with psychiatric diagnoses and healthcare workers (6). The possible association of aggressive behaviour with mental illness might drive negative public perceptions and stigmatisation of people with these mental disorders, and the mandated imposition of treatment to avert further risk of interpersonal violence might even exacerbate stigma (7). Consequently, there is an international effort to reduce coercive practices in mental health care (5, 8, 9).

Most countries have some arrangements for involuntary admission to mental health care when someone is considered to have a serious mental illness. However, countries’ judicial legislation regulating these practices varies (10). Criteria for involuntary hospitalization often involve being a danger toward others, oneself or not having the ability to consent (11). In addition to this, some countries also have arrangements for the follow-up of patients with severe mental health challenges outside the hospital setting in the form of Community Treatment Orders (12). Other forms of coercive practices are involuntary treatment during hospital admission, often in the form of medication, and practices (seclusion & restraints) aimed at handling challenging and violent behaviour. These practices also vary between countries (13). While data on coercion through official channels are usually recorded in national data in most countries, informal coercion is more challenging to track. It falls into ‘grey’ areas of unreported practices and sub-threshold forms. It would also be difficult to put all these different forms of coercion into a single data collection because of conceptual issues. The legality of formal coercion can be debated at a high ethical level, such as in the light of the principle of legal capacity and related subsidiarity. At the same time, it is widely accepted that formal coercion violates human rights. According to the Convention on the Rights of Persons with Disabilities.

CRPD (2022) (14), it must be abolished accordingly. Article 15 of the UN Convention on the Rights of Persons with Disabilities upholds the human right to freedom from torture or cruel, inhuman or degrading treatment or punishment. CRPD is a crucial instrument, calling for a shift away from substituted decision-making and coercion towards equality and non-discrimination, supported decision-making, free and informed consent, effective and meaningful participation, and community inclusion. According to WHO Guideline on mental health, human rights and legislation, essential legislative provisions for eliminating coercion in mental health services and upholding the right to free and informed consent should be created. Examples include promoting and protecting the right to free and informed consent; supporting advance planning; the provision of crisis support; the prohibition of involuntary hospitalization and treatment; and eliminating seclusion and restraint (3). However, each country has its own regulation and legislation regarding collecting data on coercion (15). Consequently, it is difficult to compare the level and consequences of coercive measures between the countries and regions of Europe. When meaningful comparisons are possible, research has also shown considerable variation in use in and between different countries and geographical areas (16, 17). Geographical variation in the use of coercive interventions represents an ethical challenge in health services (18). Further, considerable conceptual and practical difficulties exist in understanding and researching compulsion and coercion. There is, for example, a difference in how coercive interventions are measured, making it difficult to compare data from different countries and studies (19–21). According to Sashidharan et al. (2019) (22), examples of good practice in this area are limited, and there is hardly any evidence about the generalisability or sustainability of individual programmes. Lorem et al. (2014) (23) added that good clinical practice cannot be separated from the formal, moral evaluation of coercion. According to the Council of Europe, good practices may aim to reduce, prevent, or even eliminate coercive practices in mental health settings. Others will indirectly result in similar outcomes by advancing the general aim to promote voluntary mental health care and support (24). An example of good practices can be the “No Restraints” movement in psychiatric services in different European countries advocates for a shift away from traditional methods of treatment that rely heavily on physical and chemical restraints. It promotes a more humane and patient-centred approach to mental health care. The movement emphasizes the importance of empowering individuals with mental illness, respecting their autonomy, and providing them with support to live fulfilling lives in the community. This approach prioritizes therapeutic interventions, rehabilitation, and social inclusion over coercion and confinement. It aims to create environments within psychiatric facilities conducive to healing and recovery, fostering collaboration between patients and healthcare professionals. The No Restraints movement reflects a growing recognition of the dignity and rights of individuals with mental health challenges, striving to create a system that prioritizes their well-being and autonomy (25).

Several projects and attempts to reduce the use of coercive interventions have shown to be successful (24). However, these are few and thinly spread internationally, and this topic needs to be investigated more. The literature review of Gooding et al. (2018) (26) suggests some actions that should be taken to reduce coercion, like the value of recovery-oriented and trauma-informed practices; laws to reduce coercive practices, ‘peer-led’ initiatives, family- or social network-directed initiatives; crisis resolution responses in hospitals, respite centres and home-based support; advance planning to improve crisis responses; the use of non-legal ‘advocacy’; supported decision-making; low-medication or no-medication alternatives; and culturally appropriate mental health support. According to Minkovitz (27), examples of good practices would be supported decision-making, non-discriminatory conflict resolution and practical support.

In Savage et al.’s (21) systematic review of the prevalence and variability of restrictive care practice use, there are differences between geographical locations in the proportion of restrictive practices applied to mental health inpatients. However, there are variations in how these prevalence data have been defined and measured. For this reason, there is a need for more research in this area, and the absence of systematic and routinely collected data is a significant barrier to studying and understanding the nature of coercion.

Rains et al. (2019) (16) compared the annual incidence of involuntary hospitalization between 2008 and 2017 for 22 countries across Australia, Europe, and New Zealand. Annual incidence data were obtained from government sources or published peer-reviewed literature. They found that the median rate of involuntary hospitalization was 106 per 100 000 inhabitants and the range was between 58 and 150 admissions per 100,000 inhabitants. A new systematic review and meta-analysis assessing the prevalence and variability of restrictive practice use (physical restraint, seclusion and chemical restraint) in adult mental health inpatient settings also found high variation in its use across different countries (28). Understanding what can explain this variation and factors that promote involuntary hospitalizations, unlike factors that prevent them, can give us valuable information about the dynamics involved and how to prevent coercive interventions. However, to do this kind of analysis and comparison of health statistics, one has to have trustworthy and valid sources of health statistics (17, 19).

Reducing using of coercive measures to a minimum is one of the goals of an ongoing COST (European Cooperation in Science and Technology) Action called FOSTREN (Fostering and Strengthening Approaches to Reducing Coercion in European Mental Health Services). This network was created in 2020, within the platform of COST (European Cooperation in Science and Technology) (29). It is a professional and research network focusing on the issue of coercion in mental health settings. It brings together top-level experts on coercion from more than 30 countries, primarily in Europe. It was designed to establish a sustainable, multidisciplinary network of researchers, practitioners, and experts by experience focused on reducing the degree to which mental health services use coercion in hospital and community mental health services. The FOSTREN network aims to exchange international expertise from all these stakeholder groups to create an integrated framework for effective implementation. Data collection systems across Europe were discussed during network meetings, and data on coercion and other related topics were informally compared. Further discussions in the network management committee meeting (where representatives from all countries involved in the Action were present) confirmed that some European countries systematically collect data on coercion in mental health facilities. Still, others do not, providing only a part of the picture of coercion across Europe. Furthermore, several countries collect data and allow access to the general public, which allows observation and analysis of trends in its use. However, it became clear from the discussions that the collection of differentiated data on coercion was underdeveloped in most European countries.

Therefore, this study aimed to use the FOSTREN network as a basis to assemble expert opinions systematically and comprehensively regarding strategies that could be employed to promote, develop, and implement an integrated and differentiated coercion data collection system in Europe at both the national and international levels. The intended outcome of this process was to identify actions needed to improve the availability, collection, and comparability of data on coercion, considering different perceived needs across countries.

## 2 Methods

Concept mapping (30), an integrated mixed methods approach, was used to examine the diverse views of European experts on strategic actions needed to improve and systematize European coercion data collection systems. The methodology comprises generating ideas (statements/items) through focus group brainstorming guided by a study-specific prompt, then conceptual sorting and priority rating of generated statements (alternative and additional ratings may be used). Sorted data are then analysed quantitatively to map out relationships among individual statements, and cluster analysis is used to identify clusters of statements representing common aspects of the studied area.

Finally, the map and its clusters are interpreted qualitatively with rating data to aid their use in, e.g. evaluation, planning and development (31, 32). This methodology was selected to meet the study objective based on its demonstrated usefulness in integrating the input of broad expert panels to guide development and planning and its capacity to enable groups of actors to visualise their ideas around an issue of mutual interest and develop common frameworks (33, 34). The participatory, structured nature of the concept mapping process was well-suited to the complexity of integrating the diverse views of coercion and data registration system articulated by researchers from different disciplines and European countries to develop policy recommendations for strategic action in the region.

The advantage of this method is that concept mapping combines qualitative input with multivariate analysis to display how a group views a particular topic visually. Unlike purely qualitative techniques, concept mapping provides a structured approach for allowing participants to co-produce the content in focus in the study and interpret visual representations of their group perceptions (33). The same methodology was successfully used before in a similar process by another COST Action which aimed to map key concepts linked to the issue of femicide (35).

Concept mapping activities were carried out from February until November 2023 in three phases: 1) brainstorming, 2) sorting and rating, and 3) representation in maps and interpretation.

### 2.1 Brainstorming

Based on discussions with the research group and in coordination with the chair of the COST Action, we developed the following focus question to orient the brainstorming activity: “What actions are needed to improve data collection about understanding coercive measures in mental health settings in your country in terms of a) availability, b) collection and c) improving comparability between countries?”. Creating the question. We based on FOSTREN glossary, and we chose a term that addresses a wide range of coercive measures considering the broad field of clinical practice.

An online survey was conducted using software based on Lime Survey (Lime Survey Software, 2023). Some demographic data, like residence country, professional background, and current occupation, were also collected. This survey link was sent via the FOSTREN newsletter, along with instructions for the entire concept mapping process to all FOSTREN members (n=180 approximately). Participants were asked to write down as many actions as possible in response to the question, with each answer containing only one idea.

The brainstorming phase was carried out in April/May 2023. Fifty-nine members from 27 countries provided actions, which the researchers checked to eliminate duplicates and to divide complex strategies into simpler ones (**Table 1**). Forty-one actions were taken forward to the following sorting and rating phase after this process. In this step, we could not recruit participants from Belgium, Bosnia and Herzegovina, Ireland, Moldova, the Netherlands, North Macedonia, Romania, and the United Kingdom. Of those 59 participants, 33 provided information about their current clinical profession or work occupation (n = 9 nurses, n = 18 psychiatrists, n = 4 physicians, and n = 2 psychologists), whereas 33 provided information about their current (partly additional) academic profession or work occupation (n = 10 professor, n = 6 lecturers, n = 14 researcher, n = 2 both researcher and lecturer and n = 1 policy advisor).

**Table 1:** Participants in the brainstorming, sorting, and rating by country of their institutions.

	Brainstorming	Sorting and rating
Austria	2	1
Bulgaria	1	0
Croatia	1	1
Cyprus	1	0
Czech Republic	1	0
Denmark	1	0
Estonia	1	0
Finland	4	3
France	2	2
Germany	4	3
Greece	3	2
Israel	1	1
Italy	10	3
Latvia	2	1
Malta	1	0
Montenegro	1	1
Norway	7	2
Netherlands	0	3
Poland	1	0
Portugal	2	1
Slovakia	2	0
Slovenia	1	1
Spain	3	3
Sweden	1	1
Switzerland	1	2
Turkey	3	2
United Kingdom	0	1
Other (Australia)	1	2
Other (United States of America)	1	0
Other (Canada)	0	1
Total	59	37

### 2.2 Sorting and rating

The sorting and rating phase was conducted from May until September 2023, combining two different assessment methods. During one of the annual working group meetings in May 2023 in Manchester, UK, the authors presented the final list of strategies to all members participating in the meeting and explained the sorting and rating activities. Sorting activities consisted of experts organizing the 41 strategies into meaningful groups, or thematic clusters, and giving them a title. Rating activities consisted of giving each strategy two ratings for 1) its relevance to the goal of strengthening data collection systems and 2) its implementation feasibility. Each strategy was given a value from 1 to 6 for relevance and feasibility, where 1 meant very low relevance or feasibility, and 6 meant very high relevance or feasibility. Experts who still needed to finish the sorting and rating in the meeting or who were absent could complete the sorting and rating online.

In this step, 37 experts from 21 countries participated (**Table 1**). There were no participants from Belgium, Bosnia and Herzegovina, Bulgaria, Cyprus, Czech Republic, Denmark, Estonia, Ireland, Malta, Moldova, North Macedonia, Poland, Romania, and Slovakia. On the other hand, in comparison with involvement in the brainstorming phase, we additionally recruited participants from Canada the Netherlands, the United Kingdom and, countries that had not participated in the brainstorming phase. Of those 37 participants, 26 provided information about their current clinical profession or work occupation, and these were broadly comparable to the groups in the brainstorming phase (n = 10 nurse, n = 13 psychiatrist, n = 1 physician in training and n = 2 psychologists) and 20 provided information about their current (partly additional) academic profession or work occupation (n = 2 professor, n = 2 lecturer, n = 11 researcher, n = 4 both researcher and lecturer and n = 1 policy advisor).

### 2.3 Representation in maps and interpretation

In the next step of representation and interpretation, the gathered data was analyzed using concept mapping methods to identify thematic clusters and areas of consensus regarding assessed actions. After sorting and rating by participants, collected qualitative data was converted into quantitative units of data (the statement numbers and the numbers of statements connected via sorting). In mapping methodology, non-metric multidimensional scaling (MDS) is the foundational analysis that supports the distribution of ideas or items across the dimensions (32). The analysis was conducted using SPSS version 29 by IBM Statistics. Statistical procedures were conducted in line with the process described by Kane and Trochim (33).

At first, a total square similarity matrix was calculated for grouping strategies across all participants, which captures how often experts grouped them. In this step, data from 2 participants had to be excluded, as they did not group strategies according to the instructions. This matrix was then analyzed using multidimensional scaling to generate a two-dimensional point map, where strategies are plotted based on the calculated similarity measures so that strategies that were more frequently sorted together are positioned closer to each other. The stress index reflects the degree of fit between the point map and the actual data in the similarity matrix, with a lower value indicating a better fit. The final point map used as the base for hierarchical cluster analysis had a stress index of 0.28. According to Kane and Trochim (2007) (33), concept mapping projects analyzed in a meta-analysis typically achieved an average stress value of 0.285, with a range of 0.205 and 0.365 so degree of fit was acceptable.

In the hierarchical cluster analysis, the strategies were aggregated into clusters based on their proximity in the final point map. The suitability of different numbers of clusters (ranging from 2 to 9) were discussed by the research group. Initial results of possible clusters from the hierarchical cluster analysis are provided in **Supplementary Figure 1**. In the evaluation of the best number of clusters, conceptual coherence and the value of precision were considered. Seven clusters in 2 domains were identified as the ideal solution, and names were assigned after discussion within the research group. This was also reflected by the number of groups participants used within the sorting activity. Participants used an average of n = 7.0 groups (SD = 2.15, min: 3, max: 10) to sort the provided strategies. Finally, ratings of feasibility and relevance of each strategy were analyzed regarding individual strategies as well as identified clusters.

As we had a variety of countries in the sample, we wanted to explore whether the level of development of the health sector influenced the rating of presented strategies to reduce coercion. The rationale behind this assumption was that in countries with better health systems, some strategies might have a different level of relevance due to differences in the status quo. However, those strategies would also differ in feasibility due to available resources.

We chose the Legatum Prosperity Index (LPI) as a proxy for this (36). The LPI is calculated by the Legatum Institute, and their published ranking is based on various factors. It includes 9 sub-indexes ranging from 0–100, of which we used the sub score “health” for this purpose. This score was used to classify participants in two groups based on their country’s healthcare prosperity. The first category included 19 ratings from countries (Austria, Australia, Canada, Croatia, France, Greece, Latvia, Montenegro, Portugal, Slovenia, Spain, Turkey and United Kingdom) with a lower health prosperity score (below or equal to 80,5). The second category included 18 ratings from countries (Finland, Germany, Israel, Italy, Netherlands, Norway, Sweden and Switzerland) with a higher health prosperity score (above 80,5).

## 3 Results

### 3.1 Actions to promote improvement of data collection

**Figure 1:**
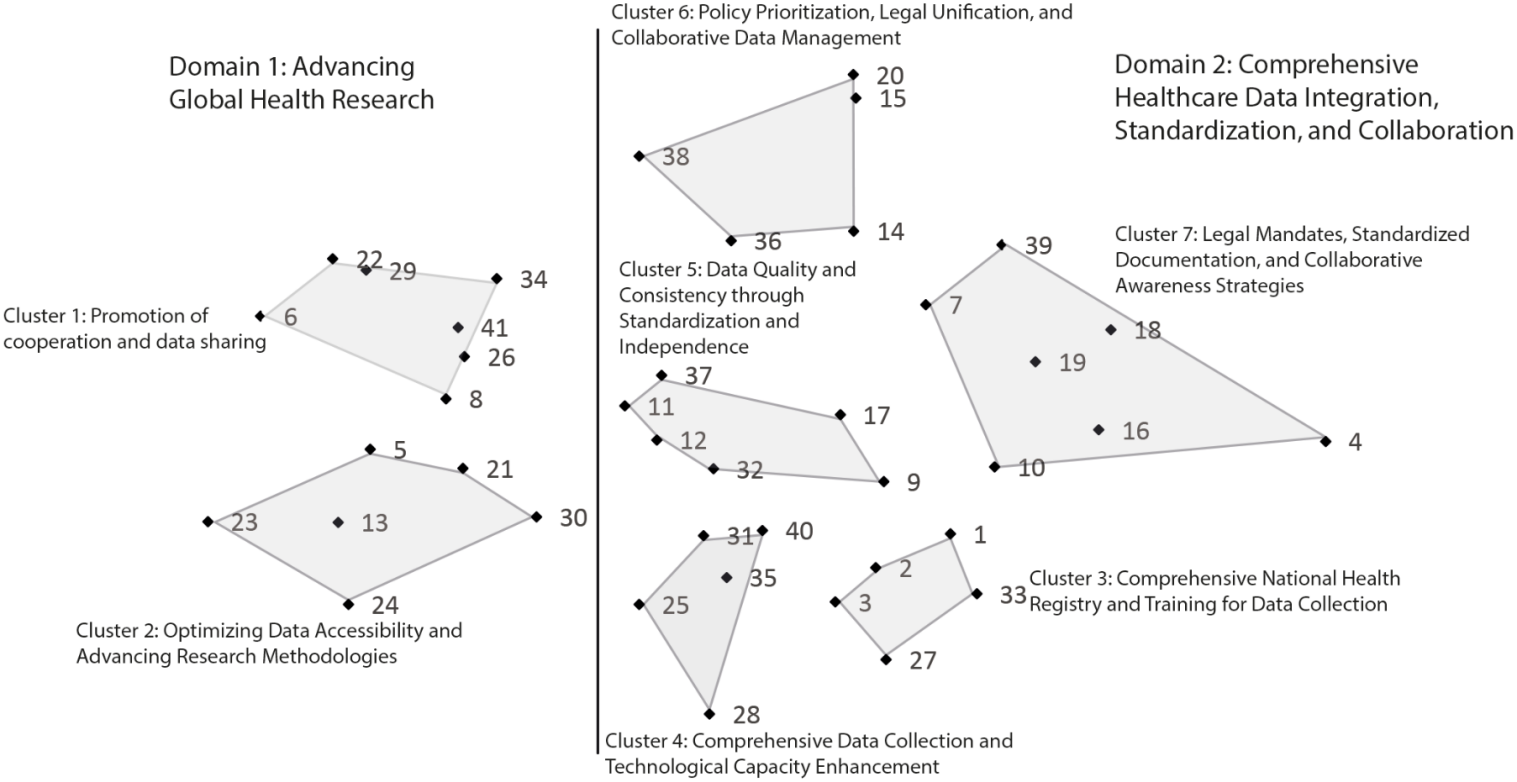
Point map of sorting strategies, including final solution of clusters and domains.

Of the 41 strategies generated by the participants in the brainstorming phase, the final clustering solution identified seven thematic clusters of possible actions: “data analysis and exchange/promotion cooperation and data sharing”, “optimizing data accessibility and advancing research methodologies”, “comprehensive national health registry and training for coercion data collection”, “comprehensive coercive data collection and technological capacity enhancement”, “quality and consistency through standardization and independence”, “policy prioritization, legal unification, and collaborative data management” and “legal mandates, standardized documentation, and collaborative awareness strategies for mental health record”. Based on the statistical relationships among these clusters and discussion within the research group, they were additionally divided into two domains: “Advancing Global Health Research: Collaboration, Accessibility, and Technological Innovations/Advancing International Research” and “Strategies for Comprehensive Healthcare Data Integration, Standardization, and Collaboration”. An overview of clusters and domains is provided in the following point map (**Figure 1**). A complete list of all strategies is provided in the **Supplementary Table 1**.

### 3.2 Identifying action with high priority

**Table 2** includes the mean ratings of relevance and feasibility for each cluster and domain. In general, relevance was rated higher than feasibility. No differences could be found regarding the two identified domains in rating of relevance (T (39) = -1.44, p = 0.080) nor feasibility (T (39) = 1.14, p = 0.130) of respective strategies in those domains. There were also no statistically significant differences between ratings of the specific strategies within the seven clusters (relevance: F(6) = 2.02, p = 0.090; feasibility: F(6) = 2.20, p = 0.067). On a descriptive level cluster 3 “Comprehensive National Health Registry and Training for Coercion Data Collection” showed the highest rated relevance and cluster 2 “Optimizing Data Accessibility and Advancing Research Methodologies” the least rated relevance. Cluster 3 “Comprehensive National Health Registry and Training for Coercion Data Collection” also showed the highest rated feasibility and cluster 6 “Policy Prioritization, Legal Unification, and Collaborative Data Management”, the least rated feasibility.

**Table 2:** Overview of identified clusters and domains from collected strategies, including mean ratings of domains and clusters on relevance and feasibility (M ± SD).

Domain (label)	Cluster (label)	Strategy (number)
1)Advancing Global Health Research: Collaboration, Accessibility, and Technological Innovations/ Advancing International Research relevance: 4.37 ± 0.48 feasibility: 4.15 ± 0.40	1)Promotion of cooperation and data sharing relevance: 4.56 ± 0.25 feasibility: 4.18 ± 0.35	22, 29, 6, 26, 41, 8, 34
	2)Optimizing Data Accessibility and Advancing Research Methodologies relevance: 4.14 ± 0.59 feasibility: 4.11 ± 0.47	5, 21, 30, 13, 23, 24
2)Comprehensive Healthcare Data Integration, Standardization, and Collaboration relevance: 4.62 ± 0.53 feasibility: 4.00 ± 0.42	3)Comprehensive National Health Registry and Training for Data Collection relevance: 5.02 ± 0.70 feasibility: 4.30 ± 0.09	1, 33, 2, 3, 27
	4)Comprehensive Data Collection and Technological Capacity Enhancement relevance: 4.19 ± 0.30 feasibility: 3.74 ± 0.28	31, 40, 35, 25, 28
	5)Data Quality and Consistency through Standardization and Independence relevance: 4.67 ± 0.30 feasibility: 4.03 ± 0.19	11, 37, 12, 32, 9, 10, 17
	6)Policy Prioritization, Legal Unification, and Collaborative Data Management relevance: 4.57 ± 0.36 feasibility: 3.64 ± 0.56	15, 20, 14, 36, 38
	7)Legal Mandates, Standardized Documentation, and Collaborative Awareness Strategies relevance: 4.61 ± 0.70 feasibility: 4.20 ± 0.48	7, 39, 18, 19, 16, 4

Furthermore, we analyzed each strategy’s mean rating of relevance and feasibility individually and the strategies with the highest and lowest ratings were as follows (Ratings for each strategy can be found in the table in the **Supplementary Table 1**. The three strategies rated with least relevance were #24 “use of artificial intelligence” (3.51 ± 1.33), #23 “use of push newsletters or apps/software to keep reviews up to date” (3.51 ± 1.41) and #39 “national template for documentation of certain multidisciplinary team meetings” (3.65 ± 1.46). On the other hand, the following strategies were rated as most relevant #2 “Collection of reliable data (all cases, all types of coercive measures, for all legal bases)” (5.68 ± 0.67), #1 “Implementation of nationwide (health) register, including data on coercive measures” (5.62 ± 0.79) and #4 “Equal understanding/definition of different coercive measures” (5.49 ± 0.77). With regard to feasibility, the following three strategies were rated lowest: #20 “Unification of different mental health laws (on a national or international level)” (3.03 ± 1.64), #36 “Mandatory report of data into a European database” (3.08 ± 1.83) and #24 “Use of artificial intelligence” (3.22 ± 1.57). On the other hand, the following strategies were rated as most feasible: #22 “Coordination of researchers, e.g. through COST networks or European initiatives” (4.76 ± 1.30), #7 “Legal obligation regarding data collection for all psychiatric institutions” (4.73 ± 1.48) and #41 “Validation of one subjective coercion scale throughout different (FOSTREN) countries” (4.51 ± 1.48).

### 3.3 Analyzing differences in priorities across countries

**Table 3** provides an overview of how these twelve strategies (the three strategies rated highest and lowest for relevance and feasibility to reduce coercion in mental health care) were related to health prosperity. As can be seen, overall ranking of ratings did not differ substantially between the two groups. Regarding ratings of relevance, differences could be found between strategies 21 (“Registration of studies/reviews/guidelines on data (collection)”) and 41 (“Validation of one subjective coercion scale throughout different (FOSTREN) countries”) as highest ranking and between the strategies 24 (“Use of artificial intelligence”) and 31 (“Data collection from several groups including service users, relatives and lawyers”) as lowest ranking.

**Table 3:** Overview of strategies rated highest and lowest on relevance and feasibility in comparison of countries with lower or higher health prosperity score (M ± SD).

		Lower health prosperity score		Higher health prosperity score	
		*Strategy number*	*Mean ± SD*	*Strategy number*	*Mean ± SD*
Feasibility rating	Most feasible	2	5.58 ± 0.84	2	5.78 ± 0.43
		1	5.58 ± 0.84	7	5.72 ± 0.83
		4	5.42 ± 0.84	1	5.67 ± 0.77
	Least feasible	24	3.68 ± 1.34	23	3.28 ± 1.53
		23	3.74 ± 1.28	24	3.33 ± 1.33
		20	3.84 ± 1.38	39	3.33 ± 1.53
Relevance rating	Most relevant	41	4.79 ± 1.34	22	4.78 ± 1.52
		22	4.74 ± 1.10	21	4.78 ± 1.70
		7	4.68 ± 1.36	7	4.78 ± 1.66
	Least relevant	36	3.16 ± 1.86	20	2.83 ± 1.65
		20	3.21 ± 1.65	31	2.89 ± 1.78
		24	3.37 ± 1.46	36	3.00 ± 1.85

## 4 Discussion

This study aimed to gather expert opinions on strategies that may be applied within and across different countries to improve the availability, collection, and monitoring of coercion data. Firstly, study participants were asked to brainstorm actions which would likely enhance the availability, collection, and comparability of data on coercion, considering the diversity of perceived needs and resources across countries. Secondly, the identified actions were sorted into groups and rated based on their relevance to strengthening data collection systems and their feasibility for implementation. In total, 41 strategies were identified and organised into seven thematic clusters.

The most relevant strategy, as rated by our participants, is the collection of reliable data in the nationwide health register, ensuring that countries share a common understanding/definition of different coercive measures. This aligns with recommendations from previous studies. Savage et al. (2024) (21) proposed a standardized set of international measures to be uniformly reported in each country, advocating for the World Health Organization to incorporate these measures into their Mental Health Atlas for international comparison. Similarly, over a decade ago, Steinert et al. (37) emphasized the need to establish common key indicators for using coercion across Europe. Our study will advance these objectives, building on initiatives undertaken by COST Action FOSTREN in recent years. For instance, the network developed a glossary to foster a shared understanding of the definitions of various coercive measures, serving as a foundation for collecting uniform data.

Our respondents did not consider the feasibility of establishing a shared European database for coercive measures to be high, nor did they envision the unification of mental health legislation. This perception may mirror the current clinical reality, which remains distant from achieving such goals. In the review by Savage et al. (2024) (21), rates of different coercive practices were explored and compared across nine countries, including five in Europe: England, Germany, Ireland, The Netherlands, and Wales. The study revealed significant variation in the types and definitions of reported coercive practices, with poor reporting overall.

Additionally, access to data varied among the countries, with England having free online access, while southwest Germany, The Netherlands, and Wales require special access to current data. Currently, reporting is diverse, and mental health legislation differs significantly between countries. Criteria for compulsive admission, patient rights during the process, and the duration of involuntary care, for example, vary (5).

Taking steps to enhance the quality and accessibility of national databases and pursuing updated legislation that aligns with the Convention on the Rights of Persons with Disabilities (CRPD) (14) could be feasible short-term actions. The strategy “Unification of mental health laws” was rated as least feasible, but the cluster “Legal Mandates, Standardized Documentation, and Collaborative Awareness Strategies” was rated high in relevance and feasibility. It means that policymakers in Europe should prioritize long-term goals with achievable milestones, such as creating shared databases.

Surprisingly, using artificial intelligence to facilitate advancements in data collection across Europe was deemed irrelevant and not feasible. Given that our respondents represent healthcare professionals and researchers in mental health, this finding may be attributed to ethical and privacy concerns (38) surrounding this susceptible topic of coercion. The level of digitalization in health care also varies among countries. According to a recent study, Finland, Denmark, the Netherlands, and Sweden are leading the EU in digitalization (39), and all of these countries are represented in our study. On the other hand, many of our respondents hail from countries with the lowest level of digitalization, primarily in Central and Eastern Europe, such as Bulgaria and Slovakia. Nevertheless, digital health care, encompassing artificial intelligence, stands out as one of the key strategic priorities in the World Health Organization’s European Regional Committee, particularly in the context of predictive analytics (40). Majcherek et al. (2024) (39) suggest that having a single European-level strategy to promote digital health may not be feasible. They recognize varying readiness levels for digitalization and advocate for different strategies based on each country’s profile.

This might also apply to our study, where a hierarchy of actions could be established based on each country’s readiness to promote, develop, and implement integrated and differentiated coercion data collection systems. For example, countries with lower readiness for digitalization could initiate actions identified as most feasible here, such as “Coordination of researchers, e.g. through COST networks or European initiatives”, “Legal obligation regarding data collection for all psychiatric institutions”, and “Validation of one subjective coercion scale throughout different (FOSTREN) countries”. On the other hand, countries with more resources in healthcare overall and a high level of digitalization could initiate efforts to create a shared infrastructure for a European database, take steps towards harmonizing mental health legislation in the region and explore how artificial intelligence, including various natural language processing models (38) could assist clinicians in reporting the use of coercion into a shared database.

Our findings not only highlight the state of data collection and monitoring of coercion use but also underscore varying capacities in providing and reporting psychiatric care for the vulnerable group of individuals in our societies, those cared for against their will. Salize and colleagues (2023) (41) reviewed forensic mental health services in 23 European countries, revealing a lack of clear definition of what constitutes a forensic bed, not to mention other national statistics informing service use and capacity. The authors concluded that stakeholders in this field lack the most basic information to describe their systems and analyze their outcomes. They advocate for a basic, minimum standardized national reporting system to inform regular EU-wide forensic psychiatry reports as a prerequisite for evaluating and comparing various systems. This approach would enable researchers, practitioners, and policymakers to identify models of best practice, effectiveness, and efficiency. Therefore, the recommendations of this study are closely aligned with the general needs of European mental health care: we must collect the data uniformly to enable comparisons and collaborative efforts which can enhance the care provided.

We find it pertinent to highlight the main limitations of this study, enabling the reader to assess the results appropriately and apply them in other contexts. Firstly, the study is cross-sectional, and any inference drawn should be considered a hypothesis. Secondly, despite the numerical significance of the responses, there is a varied distribution among the participants’ different countries of origin. Thirdly, the survey provides no insight into those alternative practices and their availability. A database or at least a systematic collection of those practices is significant, as well as the documentation of the coercive ones. Lastly, the methodology employed results in formulating a theoretical framework that requires further validation through additional studies.

With awareness of these limitations, we propose that the findings from this study have enabled an improved understanding of what is possible when striving to construct a robust dataset which could enable international benchmarking. Our sample of international experts have identified a wide range of potential actions available to health authorities committed to this goal. They have also estimated the relevance and feasibility of these actions as part of a national and international strategy to tackle this problem. A more balanced view, framed in its ethical base, must be provided in this field more than ever. This is necessary in order to avoid the risk of normalizing the use of coercive practices that are still regarded as one of the most controversial aspects of current psychiatry and mental healthcare across the world. Improved data collection on its own will not enable the implementation of coercion-free mental health services worldwide, but it is one key part of the solution and various practical steps toward achieving this goal have been identified here.

## 5 Conclusions

In conclusion, the results of this study provide a suggestion for the following steps to be taken to improve data collection and monitoring on coercion in and across European countries: advancing health research in areas of collaboration, accessibility, and technological innovations and applying the strategies for data integration standardisation, and cooperation between nations. Furthermore, expert assessments revealed that the approaches between governments and their health prosperity do not differ substantially. Respondents did not consider the feasibility of establishing a shared European database for coercive measures to be high, nor did they envision the unification of mental health legislation in the future.

According to the experts, there is a need to create and share reliable data in the nationwide health register, ensuring that countries understand different coercive measures similarly. Once in place, the evidence produced can contribute to increased public awareness and demand for a public health sector response, as done with IPV, as well as providing concrete information on risk factors and risk groups to guide police, legal, educational, and political forces in developing prevention strategies and services.

## Data Availability

The raw data supporting the conclusions of this article will be made available by the authors, without undue reservation.

## References

[1] O’brien AJ Golding CG. Coercion in mental healthcare: the principle of least coercive care. J Psychiatr Ment Health Nurs. (2003) 10:167–73. doi: 10.1046/j.1365-2850.2003.00571.x 12662333

[2] Szmukler G. Compulsion and “coercion” in mental health care. World Psychiatry. (2015) 14:259–61. doi: 10.1002/wps.20264 PMC4592637 26407770

[3] World Health Organization. United Nations (represented by the Office of the United Nations High Commissioner for Human Rights. Mental health, human rights and legislation: guidance and practice. Geneva (2023). Available online at: https://iris.who.int/bitstream/handle/10665/373126/9789240080737-eng.pdf?sequence=1.

[4] Hem MH Gjerberg E Husum TL Pedersen R. Ethical challenges when using coercion in mental healthcare: A systematic literature review. Nurs Ethics. (2018) 25:92–110. doi: 10.1177/0969733016629770 26931767

[5] Wasserman D Apter G Baeken C Bailey S Balazs J Bec C. Compulsory admissions of patients with mental disorders: State of the art on ethical and legislative aspects in 40 European countries. Eur Psychiatry J Assoc Eur Psychiatr. (2020) 63:e82. doi: 10.1192/j.eurpsy.2020.79 PMC7576531 32829740

[6] Aragonés-Calleja M Sánchez-Martínez V. Evidence synthesis on coercion in mental health: An umbrella review. Int J Ment Health Nurs. (2023) 33(2):259–80. doi: 10.1111/inm.13248 37908175

[7] Hassiotis A Almvik R Fluttert F. Coercion as a response to violence in mental health-care settings. Lancet Psychiatry. (2022) 9:6–8. doi: 10.1016/S2215-0366(21)00476-4 34921797

[8] Parliamentary Assembly. Ending coercion in mental health: the need for a human rights-based approach (2019). Available online at: https://assembly.coe.int/nw/xml/XRef/Xref-XML2HTML-EN.asp?fileid=28038.

[9] Gooding PM. Mind the gap: researching “Alternatives to coercion” in mental health care. SSRN Electron J. (2021) 273–87. doi: 10.2139/ssrn.4072170

[10] Georgieva I Whittington R Lauvrud C Steinert T Wikman S Lepping P. International variations in mental-health law regulating involuntary commitment of psychiatric patients as measured by the Mental Health Legislation Attitudes Scale. Med Sci Law. (2019) 59:104–14. doi: 10.1177/0025802419841139 30982427

[11] Brown J. The changing purpose of mental health law: From medicalism to legalism to new legalism. Int J Law Psychiatry. (2016) 47:1–9. doi: 10.1016/j.ijlp.2016.02.021 27059132

[12] O’Brien A-M Farrell SJ Faulkner S. Community treatment orders: beyond hospital utilization rates examining the association of community treatment orders with community engagement and supportive housing. Community Ment Health J. (2009) 45:415–9. doi: 10.1007/s10597-009-9203-x 19728089

[13] Bowers L Douzenis A Galeazzi GM Forghieri M Tsopelas C Simpson A. Disruptive and dangerous behaviour by patients on acute psychiatric wards in three European centres. Soc Psychiatry Psychiatr Epidemiol. (2005) 40:822–8. doi: 10.1007/s00127-005-0967-1 16172813

[14] United Nations. Convention on the rights of persons with disabilities (2006). Available online at: https://www.ohchr.org/en/instruments-mechanisms/instruments/convention-rights-persons-disabilities.

[15] Mahler L Mielau J Heinz A Wullschleger A. Same, same but different: how the interplay of legal procedures and structural factors can influence the use of coercion. Front Psychiatry. (2019) 10:249. doi: 10.3389/fpsyt.2019.00249 31105602 PMC6491953

[16] Sheridan Rains L Zenina T Dias MC Jones R Jeffreys S Branthonne-Foster S. Variations in patterns of involuntary hospitalisation and in legal frameworks: an international comparative study. Lancet Psychiatry. (2019) 6:403–17. doi: 10.1016/S2215-0366(19)30090-2 PMC6475657 30954479

[17] Hofstad T Rugkåsa J Ose SO Nyttingnes O Kjus SHH Husum TL. Service characteristics and geographical variation in compulsory hospitalisation: an exploratory random effects within–between analysis of norwegian municipalities, 2015–2018. Front Psychiatry. (2021) 12:737698. doi: 10.3389/fpsyt.2021.737698 34955909 PMC8695843

[18] Hofstad T Husum TL Rugkåsa J Hofmann BM. Geographical variation in compulsory hospitalisation – ethical challenges. BMC Health Serv Res. (2022) 22:1507. doi: 10.1186/s12913-022-08798-2 36496384 PMC9737766

[19] Hofstad T Rugkåsa J Ose SO Nyttingnes O Husum TL. Measuring the level of compulsory hospitalisation in mental health care: The performance of different measures across areas and over time. Int J Methods Psychiatr Res. (2021) 30:e1881. doi: 10.1002/mpr.1881 34033189 PMC8412230

[20] Janssen WA Van De Sande R Noorthoorn EO Nijman HLI Bowers L Mulder CL. Methodological issues in monitoring the use of coercive measures. Int J Law Psychiatry. (2011) 34:429–38. doi: 10.1016/j.ijlp.2011.10.008 22079087

[21] Savage MK Lepping P Newton-Howes G Arnold R Staggs VS Kisely S. Comparison of coercive practices in worldwide mental healthcare: overcoming difficulties resulting from variations in monitoring strategies. BJPsych Open. (2024) 10:e26. doi: 10.1192/bjo.2023.613 38205597 PMC10790218

[22] Sashidharan SP Mezzina R Puras D. Reducing coercion in mental healthcare. Epidemiol Psychiatr Sci. (2019) 28:605–12. doi: 10.1017/S2045796019000350 PMC7032511 31284895

[23] Lorem GF Hem MH Molewijk B. Good coercion: Patients’ moral evaluation of coercion in mental health care. Int J Ment Health Nurs. (2015) 24:231–40. doi: 10.1111/inm.12106 25394674

[24] . Council of Europe. Good practices in the Council of Europe to promote Voluntary Measures in Mental Health Services. France: Strasbourg Cedex (2021). Available online at: https://www.coe.int/en/web/bioethics/compendium-report-good-practices-in-the-council-of-europe-to-promote-voluntary-measures-in-mental-health-.

[25] Zanfini R Crescenti M Coreddu G Gottarelli L Linari F Ricci M. È facile smettere di legare se sai come fare: Il no restraint è un metodo di lavoro. Nuova Rassegna Studi Psichiatr. (2022) 23. Available online at: https://www.nuovarassegnastudipsichiatrici.it/volume-23/smettere-legare-no-restraint-metodo-di-lavoro.

[26] Gooding P McSherry B Roper C Grey F. Alternatives to coercion in mental health settings: A literature review. Melbourne: Melbourne Soc Equity Institute Univ Melbourne. (2018). Available online at: https://melbourne.figshare.com/articles/report/Alternatives_to_Coercion_in_Mental_Health_Settings_A_Literature_Review/21128083/1/files/37478731.pdf.

[27] Minkovitz T. Positive policy to replace forced psychiatry, based on CRPD. (2019). Available online at: https://www.chrusp.org/home/good_practices.html.

[28] Belayneh Z Chavulak J Lee D-CA Petrakis M Haines TP. Prevalence and variability of restrictive care practice use (physical restraint, seclusion and chemical restraint) in adult mental health inpatient settings: A systematic review and meta-analysis. J Clin Nurs. (2024) 33(4):1256–81. doi: 10.1111/jocn.17041 38304928

[29] Management Committee. FOSTREN – COST action C19133 (2023). Available online at: https://fostren.eu/ (Accessed March 7, 2024).

[30] Southern DM Batterham RW Appleby NJ Young D Dunt D Guibert R. The concept mapping method. An alternative to focus group inquiry in general practice. Aust Fam Physician. (1999) 28 Suppl 1:S35–40. 9988927

[31] Hagell P Edfors E Hedin G Westergren A Hammarlund CS. Group concept mapping for evaluation and development in nursing education. Nurse Educ Pract. (2016) 20:147–53. doi: 10.1016/j.nepr.2016.08.006 27591400

[32] Kane M Rosas S. Conversations about group concept mapping: applications, examples, and enhancements. 2455 Teller Road, Thousand Oaks California 91320: SAGE Publications, Inc (2018). doi: 10.4135/9781506329161

[33] Kane M Trochim W. Concept mapping for planning and evaluation. 2455 Teller Road, Thousand Oaks California 91320 United States of America: SAGE Publications, Inc (2007). doi: 10.4135/9781412983730

[34] Rosas SR. Group concept mapping methodology: toward an epistemology of group conceptualization, complexity, and emergence. Qual Quant. (2017) 51:1403–16. doi: 10.1007/s11135-016-0340-3

[35] Vives-Cases C Goicolea I Hernández A Sanz-Barbero B Gill AK Baldry AC. Expert opinions on improving femicide data collection across europe: A concept mapping study. PloS One. (2016) 11:e0148364. doi: 10.1371/journal.pone.0148364 26859885 PMC4747603

[36] Legatum Institute. The legatum prosperity index^TM^ 2023 (2023). Available online at: https://www.prosperity.com/rankings.

[37] Steinert T Lepping P Bernhardsgrütter R Conca A Hatling T Janssen W. Incidence of seclusion and restraint in psychiatric hospitals: a literature review and survey of international trends. Soc Psychiatry Psychiatr Epidemiol. (2010) 45:889–97. doi: 10.1007/s00127-009-0132-3 19727530

[38] Jeyaraman M Balaji S Jeyaraman N Yadav S. Unraveling the ethical enigma: artificial intelligence in healthcare. Cureus. (2023) 15(8):e43262. doi: 10.7759/cureus.43262 37692617 PMC10492220

[39] Majcherek D Hegerty SW Kowalski AM Lewandowska MS Dikova D. Opportunities for healthcare digitalization in Europe: Comparative analysis of inequalities in access to medical services. Health Policy. (2024) 139:104950. doi: 10.1016/j.healthpol.2023.104950 38061175

[40] World Health Organization. Regional digital health action plan for the WHO European Region 2023–2030 (RC72) (2023). Available online at: https://www.who.int/europe/publications/i/item/EUR-RC72–5.

[41] Salize HJ Dressing H Fangerau H Gosek P Heitzman J Markiewicz I. Highly varying concepts and capacities of forensic mental health services across the European Union. Front Public Health. (2023) 11:1095743. doi: 10.3389/fpubh.2023.1095743 36778562 PMC9909593

