# Corrigendum: Expert opinions on improving coercion data collection across Europe: a concept mapping study

**DOI:** 10.3389/fpsyt.2024.1464977

**Published:** 2024-08-27

**Authors:** Jakub Lickiewicz, Simone Agnes Efkemann, Tonje Lossius Husum, Tella Lantta, Luca Pingani, Richard Whittington

**Affiliations:** ^1^ Department of Psychology, Faculty of Health Sciences, Jagiellonian University Medical College, Krakow, Poland; ^2^ LWL University Hospital Bochum, Ruhr University Bochum, Bochum, Germany; ^3^ Faculty of Health Sciences, Oslo Metropolitan University, Oslo, Norway; ^4^ Department of Nursing Science, University of Turku, Turku, Finland; ^5^ Centre for Forensic Behavioural Science, Swinburne University of Technology, Melbourne, VIC, Australia; ^6^ Department of Biomedical, Metabolic and Neural Sciences, University of Modena and Reggio Emilia, Reggio Emilia, Italy; ^7^ Dipartimento ad Attività Integrata di Salute Mentale e Dipendenze Patologiche, Azienda USL-IRCCS di Reggio Emilia, Reggio Emilia, Italy; ^8^ Centre for Research and Education in Security, Prison and Forensic Psychiatry, St. Olavs Hospital, Trondheim University Hospital, Trondheim, Norway; ^9^ Institute of Mental Health, Norwegian University of Science and Technology (NTNU), Trondheim, Norway

**Keywords:** coercion, mapping methodology, mental health, coercion reduction, health policies

In the published article, there was an error in the **Acknowledgements** section. This section previously stated:

“We thank our participants for their participation in our study.”

The corrected sentence appears below:

“This article is based upon work from COST Action FOSTREN, CA19133, supported by COST (European Cooperation in Science and Technology, www.cost.eu). We thank our participants for their participation in our study.” 

The authors apologize for this error and state that this does not change the scientific conclusions of the article in any way. The original article has been updated.

