# Supplementary material for: Expert opinions on improving coercion data collection across Europe: a concept mapping study
Source: Front Psychiatry. 2024 May 29;15:1403094. doi: 10.3389/fpsyt.2024.1403094 (PMC11167108; doi:10.3389/fpsyt.2024.1403094)

Supplementary Figure 1. Dendrogram with results from the hierarchical cluster analysis regarding clusters among sorted strategies (SPSS Output).

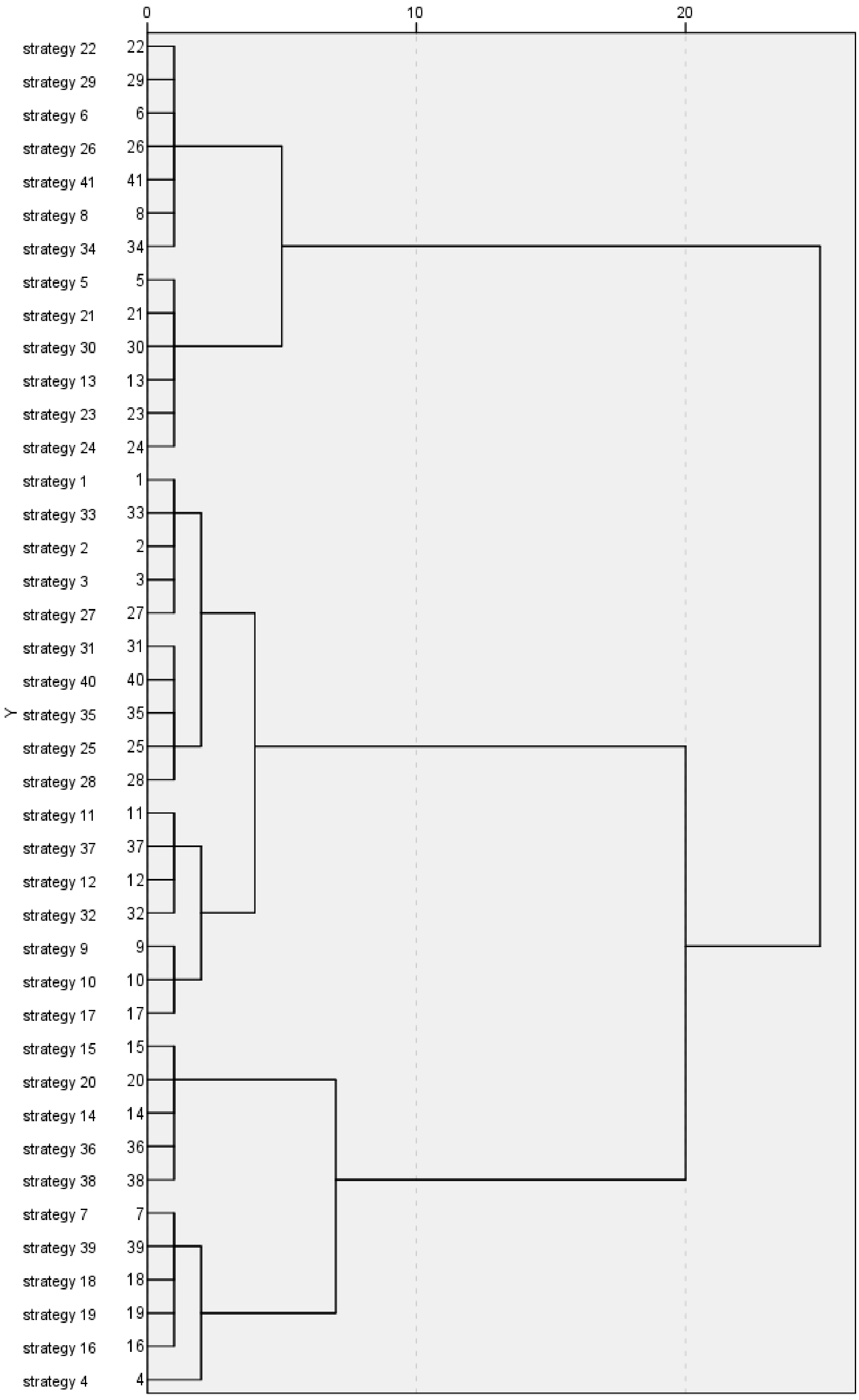

Supplement: Supplementary file 1 [file Image1.pdf]
