# Supplementary material for: Expert opinions on improving coercion data collection across Europe: a concept mapping study
Source: Front Psychiatry. 2024 May 29;15:1403094. doi: 10.3389/fpsyt.2024.1403094 (PMC11167108; doi:10.3389/fpsyt.2024.1403094)
Supplement: Supplementary file 2 [file Table1.docx]

**Supplementary Material**

Supplementary table 1. Overview of all 41 strategies including mean rating of relevance and feasibility (M ± SD)

| **Number** | **Label** | **Relevance** | **Feasibility** |
| --- | --- | --- | --- |
| 1 | Implementation of nation-wide (health) register, including data on coercive measures | 5.62 ± 0.79 | 4.38 ± 1.77 |
| 2 | Collection of reliable data (all cases, all types of coercive measures, for all legal bases) | 5.68 ± 0.67 | 4.27 ± 1.64 |
| 3 | Use of electronic patient record system (automatic data collection) | 5.00 ± 1.08 | 4.16 ± 1.82 |
| 4 | Equal understanding/definition of different coercive measures | 5.49 ± 0.77 | 4.41 ± 1.42 |
| 5 | Enhancing accessibility of (existing) data to research | 5.08 ± 0.86 | 4.11 ± 1.49 |
| 6 | Increasing (international) research on (existing) data | 4.86 ± 1.06 | 4.05 ± 1.68 |
| 7 | Legal obligation regarding data collection for all psychiatric institutions | 5.30 ± 1.37 | 4.73 ± 1.48 |
| 8 | Increasing data accessibility and data transparency of data to public | 4.68 ± 1.33 | 3.92 ± 1.55 |
| 9 | Standardization in the form of report of collected data (e.g. aggregated data, percentages, …) | 4.81 ± 1.13 | 4.22 ± 1.49 |
| 10 | Standardization of relevant context variables (hospital/patient characteristics, incidents, …) | 4.84 ± 1.14 | 4.03 ± 1.55 |
| 11 | Standardization in data analysis (e.g. key indicators, levels of analyses) | 4.68 ± 1.13 | 4.19 ± 1.45 |
| 12 | Data collection and analysis by an independent and competent institution (e.g. research) | 4.08 ± 1.55 | 3.68 ± 1.65 |
| 13 | Research on methods and improvement of data collection | 4.35 ± 1.06 | 4.46 ± 1.52 |
| 14 | Data monitoring/management by legal authority | 4.51 ± 1.33 | 4.22 ± 1.65 |
| 15 | Prioritization by health/legal authorities (improvement of national policy) | 5.05 ± 1.05 | 4.14 ± 1.49 |
| 16 | Taking geographical variation into account | 4.27 ± 1.24 | 3.95 ± 1.72 |
| 17 | Regular inspections for data collection to minimize inconsistencies | 4.46 ± 1.07 | 4.00 ± 1.62 |
| 18 | Allowing nurses to take more responsibility in documenting actual use of coercive measures | 4.22 ± 1.44 | 4.14 ± 1.62 |
| 19 | Increasing awareness among healthcare personnel for relevance of data collection | 4.76 ± 1.01 | 4.51 ± 1.33 |
| 20 | Unification of different mental health laws (on national or international level) | 4.08 ± 1.59 | 3.03 ± 1.64 |
| 21 | Registration of studies/reviews/guidelines on data (collection) | 4.05 ± 1.51 | 4.46 ± 1.39 |
| 22 | Coordination of researchers, e.g. through COST networks or European initiatives | 4.78 ± 1.20 | 4.76 ± 1.30 |
| 23 | Use of push newsletters or apps/software to keep reviews up to date | 3.51 ± 1.41 | 4.05 ± 1.60 |
| 24 | Use of artificial intelligence | 3.51 ± 1.33 | 3.22 ± 1.57 |
| 25 | Specific data collection applications | 3.76 ± 1.34 | 3.86 ± 1.40 |
| 26 | Collection and comparison of systems of data collection across countries | 4.43 ± 1.21 | 4.05 ± 1.56 |
| 27 | Logbook about coercive measures used in a ward | 3.95 ± 1.56 | 4.30 ± 1.58 |
| 28 | Raising digital/IT competence in the services, improving technical capacities | 4.05 ± 1.29 | 3.95 ± 1.29 |
| 29 | Better ways of communication about health statistics (national and international) | 4.41 ± 1.19 | 4.22 ± 1.32 |
| 30 | Guidelines developed on data collection methodologies | 4.35 ± 1.14 | 4.38 ± 1.50 |
| 31 | Data collection from several groups including service users, relatives, and lawyers | 4.51 ± 1.24 | 3.24 ± 1.62 |
| 32 | Implementation of comprehensive data collection protocols | 4.84 ± 1.01 | 3.95 ± 1.49 |
| 33 | Training and education of staff/key persons regarding data collection methods | 4.86 ± 1.25 | 4.41 ± 1.44 |
| 34 | Establishing data exchange agreements between different countries | 4.14 ± 1.34 | 3.73 ± 1.68 |
| 35 | Collection of data from units never using coercive measures | 4.24 ± 1.62 | 3.89 ± 1.65 |
| 36 | Mandatory report of data into a European database | 4.49 ± 1.52 | 3.08 ± 1.83 |
| 37 | Use of international established, validated tools/instruments | 4.97 ± 0.83 | 4.16 ± 1.40 |
| 38 | Implementation of effective cooperation between various ministries/professional groups | 4.73 ± 1.17 | 3.73 ± 1.33 |
| 39 | National template for documentation of certain multidisciplinary care team meetings | 3.65 ± 1.46 | 3.38 ± 1.64 |
| 40 | Data collection at different timepoints: before, during and after the coercive measure | 4.41 ± 1.38 | 3.76 ± 1.59 |
| 41 | Validation of one subjective coercion scale throughout different (FOSTREN) countries | 4.65 ± 1.36 | 4.51 ± 1.48 |
